# Standard pH Values for the Potassium Hydrogen Phthalate Reference Buffer Solution from 0 to 60 °C

**DOI:** 10.6028/jres.081A.005

**Published:** 1977-02-01

**Authors:** Hannah B. Hetzer, Richard A. Durst, R. A. Robinson, Roger G. Bates

**Affiliations:** Institute for Materials Research, National Bureau of Standards, Washington, D. C. 20234; Department of Chemistry, University of Florida, Gainesville, Florida 32611

**Keywords:** Acidity, emf, pH, phthalate buffer, potassium hydrogen phthalate, standard for pH

## Abstract

The standard pH values of the solution of potassium hydrogen phthalate (molality 0.05 mol kg^−1^) have been redetermined over the temperature range 0 to 60 °C, using SRM 185d. Extensive measurements were made of the emf of cells of the type

$$\text{Pd};H_{2}\left(g,\;1\;\text{atm}\right)|\text{KH phthalate}\;\left(m=0.05\right),\;\text{KCl}\;\left(m\right)|\text{AgCl};\text{Ag}$$

where *m*_KCl_ was 0.015, 0.01, or 0.005 mol kg^−1^, from which values of the acidity function p(*a*_H_*γ*_Cl_) were derived. The pH convention defines *γ*_Cl_ the range of ionic strengths 0 to 0.1 mol kg^−1^ and permits conventional values of p*a*_H_ be obtained. According to NBS procedures, p*a*_H_ for selected reference solutions is identified with the standard pH(S) in the operational definition of pH. The new values, given in terms of the thermodynamic temperature (*T*) by

$$\text{PH}\left(S\right)=\frac{2073.44}{T}-13.3270+0.045199T-3.4846\times 10^{-5}T^{2}$$

differ, on the average, by 0.003 unit from the results based on the 1944 data of Hamer and Acree.

## 1. Introduction

The NBS standard pH scale is fixed by a series of primary standards [1–3]^1^, the pH(S) values of which are based on precise emf measurements of cells without liquid junction of the type

$$\text{Pt or}\;\text{Pd};H_{2}\left(g,\;1\;\text{atm}\right)|\text{Buffer}+{\text{Cl}}^{-}|\text{AgCl};\text{Ag}$$ (A)

The pH is derived from the emf of cell *A*, together with a convention [4] relating the numerical value of the activity coefficient of chloride ion to the ionic strength (*I*) and the temperature, where 0 < *I* ⋜ 0.1. It has been shown [3] that, over the pH range 3.5 to 9.2, this scale is internally consistent within 0.006 unit at 25 °C and within 0.013 unit at 10 and 40 °C; that is to say, it is immaterial, within these limits, which of the standards is chosen for the calibration of pH measuring equipment in a given situation.

The solution of potassium hydrogen phthalate, molality 0.05 mol kg^−1^, is probably the most commonly used of the NBS primary standard reference solutions. It is likewise the single primary standard of the British Standard Institution’s pH scale [5]. This solution is easy to prepare from readily available certified material and is reasonably stable. Furthermore, its pH is not markedly sensitive to temperature changes and lies in the weakly acidic region where the practical measurement and control of acidity is often required.

The precise emf measurements, on which the NBS values for this important standard reference solution were originally based, were reported by Hamer, Pinching, and Acree between 1944 and 1946 [6–9]. The potassium hydrogen phthalate used in their work consisted of Standard Reference Materials 84a and 84b. These lots, certified for acidimetry, assayed very close to 100.00 percent. No extensive redetermination of pH(S) over the range 0 to 60 °C was undertaken preparatory to the certification of potassium hydrogen phthalate, SRM 185, as a pH standard, but it was shown [9] that 0.05 *m* solutions prepared from lots 84a, 84b, 84c, and 185 had the same pH value. The certification of three subsequent lots (SRM 185a, 185b, and 185c) was made by comparison, usually at 25 °C, of the emf of cell *A* found for the 0.05 molal phthalate solution, with added KCl, with corresponding data obtained in the earlier extensive investigation. The earlier values of pH(S) were then altered uniformly over the temperature range by an amount corresponding to the difference of emf found, at most a few thousandths of a pH unit. It is the purpose of this paper to report the results of a thorough study conducted prior to the certification of SRM 185d, a lot of which was shown by careful assay to have a purity of 99.99 percent and therefore presumably to be essentially identical with the material used in the earlier investigations of Hamer et al.

## 2. Method

The acidity function p(*a*_H_*γ*_Cl_) is readily calculated from the emf (*E*) and standard emf (*E*^0^) of cell *A* by the equation

$$p\left(a_{H}\gamma_{\text{Cl}}\right)\equiv -\log\left(m_{H}\gamma_{H}\gamma_{\text{Cl}}\right)=\frac{\left(E-E^{0}\right)F}{RT\;\ln\;10}+\log\;m_{\text{Cl}}$$ (1)

the molality of chloride ion (*m*_Cl_) being known. As in the later work on the establishment of standard reference buffer solutions [10], three molalities of KCl, namely 0.015, 0.01, and 0.005 mol kg^−1^, were used. The values of p(*a*_H_*γ*_Cl_) decreased linearly as *m*_KCl_ increased, and the intercepts p(*a*_H_*γ*_Cl_)^0^ at *m*_KCl_ = 0 were determined by linear regression analysis:

$$p\left(a_{H}\gamma_{\text{Cl}}\right)=p{\left(a_{H}\gamma_{\text{Cl}}\right)}^{0}-bm_{\text{KCl}}$$ (2)

The final step in the assignment of standard values, pH(S), to the chloride-free phthalate buffer solution was the calculation of p*a*_H_ from p(*a*_H_*γ*_Cl_)^0^:

$$\text{pH}\left(S\right)\equiv pa_{H}=p{\left(a_{H}\gamma_{\text{Cl}}\right)}^{0}+\log\;\gamma_{\text{Cl}}$$ (3)

where *γ*_Cl_ is fixed by the pH convention proposed by Bates and Guggenheim [4]:

$$\log\;\gamma_{\text{Cl}}=\frac{-AI^{1/2}}{1+1.5I^{1/2}}$$ (4)

In eq (4)*A* is the Debye-Hückel slope constant (molality scale) for the temperature in question [3]. This convention is intended to apply over the full temperature range but is restricted to ionic strengths (*I*) no greater than 0.1 mol kg^−1^.

## 3. Experimental Procedures

In general, the experimental methods, including the preparation of the thermal-electrolytic silver-silver chloride electrodes [3] and the techniques of the emf measurements [11], were the same as those described in detail in other publications. Departures from these standard procedures are set forth below.

The platinum bases for the hydrogen electrodes must be coated with palladium black instead of platinum black, in order to minimize reduction of phthalate [12]. Preliminary measurements showed, however, that lightly coated electrodes were more stable than those with a heavier coat of palladium black. Accordingly, the platinum foils were electrolyzed at a current of about 30 mA cm^−2^ for 5 to 10 seconds in a 1 percent solution of palladium chloride acidified with HCl and containing 0.08 g of lead acetate trihydrate per 100 ml of plating solution. A strip of palladium metal served as anode.

Unfortunately, hydrogen electrodes prepared in this way are not as reproducible as are those coated with platinum black (when they can be used), and some instability at the higher temperatures gave testimony to a certain amount of reduction of phthalate. It was therefore necessary to adopt criteria for the rejection of a part of the cell data obtained in this extensive investigation. Each of the cells contained two electrodes of each kind, and the emf was measured both at the beginning and at the end of each temperature series. In general, the criteria for rejection were (a) differences greater than 0.1 mV between pairs of electrodes in the same cell, and (b) differences greater than 0.3 mV between the initial and final measurements at 25 °C. Although some of the cells behaved acceptably over the entire 0 to 60 °C temperature range, for best results it was found necessary to use fresh electrodes and solutions for the range 40 to 60 °C. Thus, the results of only 3 cells out of 27 measured at 10 °C were rejected, whereas 25 of 44 were rejected at 50 °C.

The standard emf *E*^0^ of cell *A* has been determined in two extensive series of measurements [13, 14]. Inasmuch as there is some evidence that Ag/AgCl electrodes prepared in different laboratories by slightly differing procedures have slightly different potentials, it has been recommended [15] that *E*^0^ be, in effect, redetermined under each set of experimental conditions by measurement of the emf of cell *A* containing hydrochloric acid, molality 0.01 mol kg^−1^. Inasmuch as there is substantial agreement that the mean ionic activity coefficient (molality scale) of HCl has the values 0.904 at 25 °C and 0.908 at 0 °C in this solution [15], *E*^0^ can be derived from these emf measurements.

Four investigators, namely V. E. Bower, R. Gary, H. B. Hetzer, and M. Paabo, prepared electrodes from materials used in their own studies, and the group prepared five cells from the same solution of HCl, measuring the emf at three temperatures. The mean results for *E* were 0.46102 V (standard deviation 0.00004) at 10 °C, 0.46425 V (s.d. 0.00010) at 25 °C, and 0.46623 V (s.d. 0.00005) at 40 °C, from which *E*^0^ is 0.23153, 0.22244, and 0.21216 V at 10, 25, and 40 °C.

These results are higher than those found by Bates and Bower [14]; the differences are 0.11, 0.09, and 0.08 mV at 10, 25, and 40 °C, respectively. Hence, for the present study, *E*^0^ at all temperatures from 0 to 60 °C was obtained by adding 0.1 mV to the values given in the fifth column of table 1, reference [14].

The potassium hydrogen phthalate, SRM 185d, had an assay value of 99.99 percent. It was shown to be equivalent to SRM 84a, one of the lots used by Hamer et al. [6–9], by measuring the emf of a cell of type *A* containing 0.05m KH phthalate (SRM 84a) + 0.01m KCl at 25, 40, 45, 50, 55, and 60 °C. The emf at these six temperatures differed on the average by only 0.07 mV from the mean values found in the present investigation.

## 4. Results

The emf data from the cells meeting the criteria outlined above were corrected to 1 atm hydrogen pressure. They are numerous, and to conserve space the individual values (means of two pairs of electrodes in the same cell) have been averaged and the results summarized in table 1, together with the number of cells and the mean deviation from the mean emf, in mV. Only the initial results at 25 °C are given; the final results at this temperature were not used.

Values of the acidity function p(*a*_H_*γ*_Cl_) were calculated by eq (1) and the intercepts p(*a*_H_*γ*_Cl_)^0^ and slopes *b* obtained by linear regression analysis; the relationship is shown in eq (2). This procedure was carried out in two ways: first, by use of the average p(*a*_H_*γ*_Cl_) values computed from the average emf’s of table 1, and second, by use of the entire population of p(*a*_H_*γ*_Cl_) values, that is, those derived from the emf of each individual cell. The intercepts obtained by the two procedures differed by considerably less than 0.001 pH unit. The results collected in table 2 are nonetheless those obtained by treating each data point individually. The standard deviations of the intercepts and slopes are given, and the standard deviation for regression, expressed in mV, appears in the last column.

Application of the convention for *γ*_Cl_ set forth in eq (4) enables one to derive pH(S) from p(*a*_H_*γ*_Cl_)^0^ by eq (3). The ionic strength (*I*) required for this purpose was calculated from the ionic compositions of potassium hydrogen phthalate solutions; these have been obtained by Hamer, Pinching, and Acree [9] from *K*_1_ and *K*_2_ for phthalic acid. The ionic strength is nearly constant over the temperature range 0 to 60 °C, varying only from 0.0532 to 0.0534 mol kg^−1^; log *γ*_Cl_ decreases from −0.0843 at 0 °C to −0.0934 at 60 °C. The values of pH(S) found in this way are listed in the third column of table 3. With the aid of the OMNITAB computer program, they were fitted to the equation

$$\text{pH}\left(S\right)=\frac{2073.44}{T}-13.3270+0.045199T-3.4846\times 10^{-5}T^{2}$$ (5)

where *T* is the thermodynamic temperature. Calculated values of pH(S) are given in the last column of the table. From a comparison of the experimental pH(S) with that calculated by eq (5), there is a standard deviation for regression of 0.0010 unit.

## 5. Discussion

The values of pH(S) labeled “NBS” in table 3 were derived [1] from the emf measurements of Hamer and Acree [6]. The original emf data, reported in international volts, were corrected to the absolute scale and p(*a*_H_*γ*_Cl_) obtained with the aid of *E*^0^ from the study of Bates and Bower [14]. The pH(S) values resulting from the application of the *γ*_Cl_ convention to p(*a*_H_*γ*_Cl_)^0^ were fitted to a four-constant equation of the same form as eq (5). The mean difference between experimental and calculated pH(S) at the 13 temperatures was 0.002_1_. The smoothed values from this equation are listed in the second column of table 3. In general, the present results for temperatures below 20 °C are slightly higher than those derived from the earlier data, and they are lower when the temperature lies between 20 and 60 °C. Nevertheless, the mean difference for all 13 temperatures is only 0.003_3_ unit.

In 1946, Hamer, Pinching, and Acree [9] calculated the p*a*_H_ values of solutions of potassium hydrogen phthalate at eight different molalities, 0.001 to 0.2 mol kg^−1^, from *K*_1_ and *K*_2_ for phthalic acid and ionic activity coefficients estimated in a manner different from the convention of eq (4). At a molality of 0.05 mol kg^−1^, the values obtained in this way are in somewhat better agreement with those of the present study than are the values labeled “NBS” in table 3. The mean difference is 0.002_2_ at the 13 temperatures; if 55 and 60 °C (where the differences are 0.006 and 0.010 pH unit, respectively) are omitted, the mean difference is only 0.001 unit.

The largest differences between the values in the second and third columns of table 3 occur at 0 and 35 °C. In this connection, it is noteworthy that the experimental value from the earlier work is 4.006 at 0 °C; here smoothing has accentuated the discrepancy. Furthermore, the values of the present study for the range 40 to 60 °C are based largely on a different set of cells and solutions from those for 35 °C and below. The greatest disparity between experimental and calculated pH(S), namely 0.0024 unit, occurs at 35 °C, and it seems likely that the experimental result at this temperature is slightly low. No explanation for this inconsistency can be found, yet its existence appears to be sufficient justification to regard the smoothed values of the last column as the more reliable and self-consistent set.

## Figures and Tables

**Table 1:** Electromotive force of the cell: *Pd*; *H_2_* (g, 1 atm) |*KH* Phthalate (m = 0.05), *KCl* (m) | *AgCl*; *Ag* for m*_KCl_* = 0.005, 0.01, and 0.015 mol kg^−1^ from 0 to 60°C (in volts)

*t/°*C	*m*_KCl_ = 0.005			*m*_KCl_ = 0.01			*m*_KCl_ = 0.015		
	No. of cells	*E*/volts (mean)	Mean dev.*a*	No. of cells	*E/*volts (mean)	Mean dev.*a*	No. of cells	*E/*volts (mean)	Mean dev.*a*

0	6	0.58311	0.03	8	0.56662	0.07	10	0.55695	0.09
5	6	0.58666	0.04	8	0.56986	0.06	10	0.55996	0.07
10	6	0.59015	0.03	8	0.57304	0.06	10	0.56297	0.07
15	6	0.59366	0.05	8	0.57622	0.07	10	0.56601	0.08
20	6	0.59713	0.06	8	0.57939	0.06	10	0.56900	0.08
25	12	0.60060	0.06	12	0.58257	0.04	17	0.57199	0.09
30	6	0.60402	0.09	8	0.58565	0.07	10	0.57493	0.10
35	6	0.60742	0.13	8	0.58873	0.06	11	0.57786	0.12
40	6	0.61104	0.15	6	0.59209	0.05	8	0.58090	0.08
45	6	0.61444	0.12	6	0.59519	0.07	7	0.58385	0.13
50	6	0.61787	0.09	6	0.59832	0.10	7	0.58680	0.13
55	6	0.62131	0.09	6	0.60144	0.10	7	0.58969	0.15
60	6	0.62474	0.08	6	0.60460	0.10	7	0.59266	0.12

a Mean deviation from the mean value of *E*, in mV.

**Table 2:** Linear regression analysis. Constants of equation (2)

*t*/*°* C	*n*	p(*a*_H_*γ*_Cl_)°	*b*	s.d. (intercept)	s.d. (slope)	s.d. (regression)*a*

0	24	4.0944	0.612	0.0006	0.049	0.05
5	24	4.0880	0.659	0.0008	0.070	0.08
10	24	4.0857	0.669	0.0008	0.071	0.08
15	24	4.0858	0.630	0.0009	0.082	0.09
20	24	4.0881	0.627	0.0010	0.083	0.10
25	41	4.0946	0.654	0.0006	0.054	0.08
30	24	4.1005	0.641	0.0012	0.101	0.12
35	25	4.1075	0.612	0.0013	0.115	0.13
40	20	4.1223	0.788	0.0012	0.108	0.12
45	19	4.1341	0.746	0.0014	0.128	0.15
50	19	4.1482	0.749	0.0013	0.121	0.13
55	19	4.1638	0.858	0.0016	0.144	0.17
60	19	4.1803	0.818	0.0013	0.116	0.14

a Expressed in mV.

**Table 3:** pH(S) values for the potassium hydrogen phthalate solution, molality = 0.05 mol kg^−1^, from 0 to 60°C

*t*/*°*C	NBS*a*	Present Work	
		exp.	calc.*b*

0	4.003	4.010_1_	4.0100
5	3.999	4.003_1_	4.0035
10	3.998	4.000_2_	4.0001
15	3.999	3.999_6_	3.9994
20	4.002	4.001_2_	4.0014
25	4.008	4.007_0_	4.0058
30	4.015	4.012_1_	4.0123
35	4.024	4.018_4_	4.0208
40	4.035	4.032_4_	4.0311
45	4.047	4.043_3_	4.0431
50	4.060	4.056_6_	4.0565
55	4.075	4.071_4_	4.0712
60	4.091	4.086_9_	4.0872

a From the emf measurements of Hamer et at. [6] ; see reference [1].

b Calculated with use of eq (5).

## Footnotes

1 Figures in brackets indicate the literature references at the end of this paper.
